# Transcutaneous sentinel lymph node detection in skin melanoma with near-infrared fluorescence imaging using indocyanine green

**DOI:** 10.1097/CMR.0000000000000994

**Published:** 2024-08-02

**Authors:** Bo E. Zweedijk, Antonius W. Schurink, Thijs van Dalen, Tessa M. van Ginhoven, Cornelis Verhoef, Bernd Kremer, Denise E. Hilling, Stijn Keereweer, Dirk J. Grünhagen

**Affiliations:** a Department of Surgical Oncology and Gastrointestinal Surgery, Erasmus MC Cancer Institute; b Department of Otorhinolaryngology, Head and Neck Surgery, Erasmus MC Cancer Institute, Rotterdam, the Netherlands; c Department of Surgery, Leiden University Medical Center, Leiden, the Netherlands

**Keywords:** fluorescence imaging, fluorescence-guided surgery, indocyanine green, melanoma, molecular imaging, optical imaging, sentinel lymph node, sentinel lymph node biopsy

## Abstract

The aim of the study is to assess whether indocyanine green (ICG) fluorescence can replace technetium in the preoperative detection of sentinel lymph nodes (SLN) from cutaneous melanoma. The current golden standard for SLN detection is the radioisotope technetium. A promising alternative is fluorescence imaging (FLI) using ICG. In this study, we enrolled patients undergoing sentinel lymph node biopsy (SLNB) for skin melanoma at the Erasmus Medical Center between November 2022 and July 2023. The SLNB procedure was performed as a standard of care. After general anesthesia, ICG was injected intradermally around the primary tumor site. Both the patient and the surgeon were not blinded for the location of the SLN. FLI was performed before incision, in vivo after incision, and ex vivo. Fluorescent SLNs were confirmed using the gamma probe in all cases. Thirty-two patients were included in this study, and a total of 39 SLNs were harvested. The transcutaneous detection rate of ICG was 21.9%. The combined ex vivo ICG fluorescence and technetium uptake was 94.9%. One SLN contained only ICG (2.6%) and one SLN contained only technetium-uptake (2.6%). FLI using ICG resulted in a relatively low transcutaneous detection, which means that exclusive use of this technique in its present form is not feasible. However, we did find a high accumulation of ICG in the SLN, indicating the potential of ICG in combination with other imaging techniques.

## Introduction

Accurate staging plays a crucial role in determining appropriate treatment measures in the context of melanoma, a highly aggressive form of skin cancer known for its ability to spread at an early stage [1,2]. The sentinel lymph node biopsy (SLNB) method has emerged as a crucial component in the staging of this disease [3–6].

Traditionally, SLNB has been dependent on technetium (Tc)-99 m as a radiotracer for the purpose of lymphatic mapping [7]. Although high rates of SLN localization can be achieved (98–99%), this approach has logistical obstacles, including the requirement for a substantial time interval between the injection of the radiotracer and the surgical procedure, as well as the need for strict adherence to radiation safety guidelines [8–10]. The utilization of indocyanine green (ICG) for SLNB has experienced an increase in interest and activity in the past few years [11–17]. ICG, a fluorescent dye, emits light in the near-infrared (NIR) region after excitation using light with the correct wavelength [18]. NIR camera systems are required to detect this signal, as the human eye is not sensitive to light in the NIR region [19]. Multiple camera systems are commercially available in the operating room to detect NIR fluorescence, each with its own sensitivity and dynamic ranges for NIR fluorophores [20]. Previous studies have demonstrated a concurrent accumulation of Tc and ICG in the SLN [16,17,21–23]. In addition, the lymphatic drainage of ICG enables detection of its progression through the lymphatic vessels to the SLNs using the earlier mentioned NIR camera systems [24,25]. This, in combination with the high accumulation in the SLNs, makes ICG a potential viable substitute for Tc-based detection methods. Several studies have already demonstrated that fluorescence-guided surgery (FGS) using ICG serves as a suitable alternative for SLNB in breast cancer patients [26–28]. Whether ICG could be of additional value in melanoma surgery and eventually completely replace Tc depends on the depth of the SLNs because ICG has a penetration depth of up to 1 cm.

The utilization of ICG and Tc as dual tracers (Tc-ICG dual tracer), has been established as a standard of care for patients with head and neck melanoma at the Erasmus University Medical Center (Rotterdam, The Netherlands). Within this dual tracer, ICG and Tc are bound to each other. Tc in combination with a single-photon emission computed tomography (SPECT) scan is utilized for the general localization of SLNs, whereas ICG is used for intraoperative localization in the late operative stage [29]. Using the above method instead of only Tc results in improved (i.e. more accurate) localization of head and neck SLNs [30]. Of note, the use of this dual-tracer approach effectively tackles the particular obstacles presented by the small size and anatomical positioning of lymph nodes inside the head and neck region [31].

Our study aims to explore a more efficient, patient-friendly, and more environmentally friendly SLNB strategy for skin melanoma, with the goal of optimizing surgical approaches and reducing dependence on Tc. We will test different camera systems and ICG concentrations, in which we aim to optimize the SLN transcutaneous detectability. The primary focus is transcutaneous SLN identification.

## Methods

### Participants

This study was conducted in patients undergoing SLNB for malignant cutaneous melanoma at the Erasmus Medical Center (Rotterdam, the Netherlands) from November 2022 to July 2023. The study was approved by the medical ethical committee of the Erasmus Medical Center and was conducted in full compliance with the principles of the Declaration of Helsinki (10^th^ version, Fortaleza, 2013) and ICH GCP guidelines.

Patients younger than 18 years or patients with a known allergy to ICG or iodine were excluded. The study did not meet the criteria for the Medical Research Involving Human Subject Act (non-WMO), as ICG is a widely used, FDA approved, fluorescent agent that is already part of the standard of care [32]. No informed consent was collected.

Patient’s characteristics, such as age, gender, and BMI were collected. In addition, tumor details and histopathological status of the excised lymph nodes were recorded. All personal data was collected by the primary caregiver and was passed on to the researchers anonymously.

### Study design

Preoperative lymphoscintigraphy or SPECT scan was conducted as standard of care. SPECT was performed in the case of melanomas in the head and neck region, and lymphoscintigraphy was performed in all other melanoma locations. In case the preoperative lymphoscintigraphy or SPECT did not show SLN(s), no SLNB was performed. As the location of the SLN was marked on the skin, both the patient and surgeon were not blinded for the location of the SLN. After the preoperative imaging, patients were brought to the operating room.

After general anesthesia, and before sterile draping, ICG (Verdye) was injected intradermally around the primary tumor site by one of the researchers, followed by massaging for approximately 5 min. We used three different dosages (2.5, 5, or 10 mg), based on existing literature on SLN detection using ICG [28,33]. For melanomas located in the head and neck region, the Tc-ICG dual tracer was used as standard of care. Within this dual tracer, the dose of ICG varied from 0.01 mg to 0.04 mg. An additional dose of ICG was administered during surgery unless the surgeon preferred not to. The patient was not subjected to unnecessary harm, as decision-making was based on Tc throughout the entire procedure, in accordance with the gold standard for SLN detection. The administration of blue dye is not considered standard of care at our institution and is not preferred by our surgeons.

Fluorescence imaging (FLI) was performed using the Quest Spectrum Platform (Quest Medical Imaging, Middenmeer, Netherlands), the Visionsense VS3 HD3D camera, or a combination of both cameras. We used these different camera systems in an attempt to replicate a previous study on the same subject [28]. First, before incision, transcutaneous detection was evaluated using FLI. If there was detection of an SLN, the location was confirmed using the gamma probe. The location of the incision was based on the signal using the gamma probe. Even when the fluorescence signal was observed at a different location, or no fluorescence signal was detected at all, the incision was based on the gamma probe results. During the procedure, after incision, FLI and the gamma probe were used to find and excise the SLN. The use of FLI during the surgical procedure was not standardized. After the procedure, FLI was performed to confirm the *ex vivo* fluorescence. All excised lymph nodes were also validated using the gamma probe. Both FLI and the gamma probe were used to evaluate if there was any residual signal *in vivo*. To minimize any potential interobserver variability, the complete FLI procedure, as well as the interpretation of the images, was performed under the direct supervision of dedicated and experienced researchers (A.S., B.Z.). Therefore, the involved surgeons did not need experience in FLI and were not trained.

### Statistical analysis

Transcutaneous detection of the SLN was defined as a fluorescence spot seen on the skin that corresponded with a positive signal using the gamma probe, with the spot remaining fluorescent after incision. The transcutaneous detection rate was defined as the proportion of patients in whom at least one SLN was detected transcutaneously using the fluorescent signal of ICG. The pathological detection rate was defined as the proportion of tumor-positive SLNs detected using the fluorescent signal of ICG compared with the total number of tumor-positive SLNs.

Baseline demographics, such as BMI, age, and gender, and outcome variables were summarized using descriptive statistics. Continuous variables were presented as means with SD. Dichotomous and categorical data were presented as frequencies with percentages. Proportions were presented with the 95% confidence intervals (CI) or the interquartile range (IQR). All statistical analyses were performed using IBM SPSS Statistics (Version 28.0.1.0; Armonk, New York, USA)

## Results

### Study population

Thirty-two consecutive patients scheduled for an SLNB for malignant skin melanoma were included in this study. The patients consisted of 15 women and 17 men, with a median age of 57.0 years (IQR, 48.5–67.85), mean BMI of 27.3 kg/m^2^ (95% CI, 25.8–28.8) and all Caucasian. Most of the SLNs were located in the axillary and cervical basin [both *n* = 9 (28.1%)]. Other locations included the groin (*n* = 8, 25.0%), pre- or retro-auricular (*n* = 5, 15.6%), and ‘other’ [*n* = 1 (trunk, not in a classical lymph node basin), 3.1%]. In four patients, a second node basin was present, and in all of them, the SLNs contained ICG. For an overview of the transcutaneous detection rate per SLN location, we refer to Table 1 of our supplementary data (Supplemental Digital Content 1, http://links.lww.com/MR/A401).

**Table 1:** Patient and disease characteristics of patients with and without transcutaneous detection

	Transcutaneous detection	No transcutaneous detection
Patient characteristics		
Number of patients	7	25
Sex (% men)	57%	52%
BMI, mean (95% CI)	26.7 (24.0–29.5)	27.5 (25.7–29.3)
Age (years), median (IQR)	53.0 (41.0–76.0)	58.0 (51.0–67.5)
Disease characteristics		
Number of SLNs	8	31
Tumor-positive SLNs, *n* (%)	1 (12.5%)	6 (19.4)
Tumor burden (mm), mean (range)	9.5	1.7 (0.4–4.0)

CI, confidence interval; IQR, interquartile range; SLN, sentinel lymph nodes.

### Administration of indocyanine green

Twenty-four patients received a fixed dose of ICG peroperatively, of which eight received this dose in addition to the Tc-ICG dual tracer. The fixed dose of ICG was either 2.5 mg (*n* = 1), 5.0 mg (*n* = 8), or 10 mg (*n* = 6). In case of a combination of the dual tracer and ICG, the additional dose of ICG was either 5.0 mg (*n* = 2), or 10 mg (*n* = 7). In eight patients receiving the Tc-ICG dual tracer, no additional ICG was injected due to surgeon’s preference. No ICG-related hypersensitivity reactions or other adverse events were observed.

### *In vivo* fluorescence imaging

**Fig. 1:**
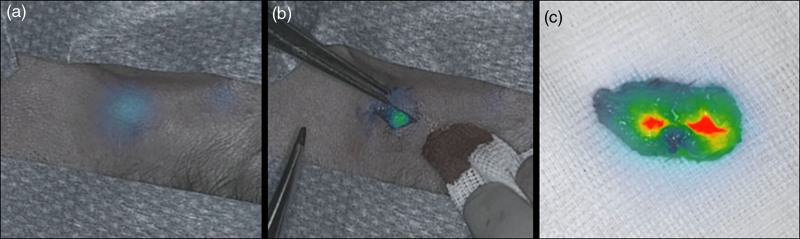
Intraoperative images of the fluorescent signal as captured by the Quest. (a) Transcutaneous fluorescent signal. (b) Fluorescent signal during the procedure. (c) ex vivo fluorescent signal.

In seven out of the 32 patients (21.9%), at least one SLN was detected transcutaneously. Figure 1 shows an example of transcutaneous detection of a SLN in the head and neck region. Table 1 shows all patient and disease characteristics of the patients with and without transcutaneous detection. In two patients, there was a mismatch between the fluorescent signal and the signal of the gamma probe, after which the incision was based on the findings of the gamma probe.

In three patients, no SLN was found during surgery using either FLI or the gamma probe. In these cases, no SLN was removed. *Ex vivo* FLI detected at least one SLN in 28 out of the 29 patients (96.6%) in whom SLN(s) were harvested.

### *Ex vivo* fluorescence imaging

A total of 39 SLNs were harvested. Of these 39 lymph nodes, 38 (97.4%) were ICG-fluorescent, and 38 (97.4%) were Tc-positive (Table 2). Combined ICG-fluorescence and Tc-uptake was observed in 37 lymph nodes (94.9%). This resulted in the mean identification of 1.31 SLNs per patient using ICG. We also evaluated the correlation between ICG and Tc in the nodes of patients who did not receive the Tc-ICG dual tracer, as within this tracer, ICG and Tc are bound to each other. This results in a one-on-one correlation between ICG and Tc positivity. When we excluded these patients, 17 out of 18 nodes (94.4%) appeared to be both ICG and Tc positive. Furthermore, one SLN only showed ICG-fluorescence uptake (2.6%) and one SLN only showed Tc-uptake (2.6%).

**Table 2:** Concordance rates of sentinel lymph node mapping per excised lymph node sent for pathology (*n* = 39)

	Tc+ *n*(%)	Tc− *n*(%)	Total *n*(%)
ICG+	37 (94.9)	1 (2.6)^a^	38 (97.4)
ICG−	1 (2.6)	NA	1 (2.6)
Total	38 (97.4)	1 (2.6)	39 (100)

ICG, indocyanine green Tc, technetium.

a One lymph node negative for technetium contained metastasis.

### Correlation between final histopathology and fluorescence imaging

After pathological examination, seven SLNs (15.2%) contained metastases (>0.1 mm). All seven tumor-positive SLNs showed ICG-uptake, resulting in a pathological detection rate of 100%. Moreover, one tumor-positive SLN did not show any Tc-uptake (2.2%). The one Tc-negative, ICG-positive, tumor-positive SLN contained a metastasis of 2.2 mm. In addition, seven regular (nonsentinel) lymph nodes were removed in order to reach the SLN. None of these lymph nodes were positive for either Tc or ICG. Furthermore, six cases of clinically nonlymphatic tissue (i.e. fatty tissue) were removed as part of the study because of a positive fluorescent signal. Both the excised regular lymph nodes and nonlymphatic tissues were free of tumor. For an overview, see Table 3.

**Table 3:** Pathology results of all lymph nodes

	ICG+/Tc+ *n*(%)	ICG+/Tc− *n*(%)	ICG−/Tc+ *n*(%)	ICG−/Tc− *n*(%)	Total *n*(%)
No metastasis	31 (67.4)	0	1 (2.2)	7 (15.2)	39 (84.8)
Metastasis	6 (13.0)	1 (2.2)	0	0	7 (15.2)
Total	37 (80.4)	1 (2.2)	1 (2.2)	7 (15.2)	46 (100)

ICG, indocyanine green; Tc, technetium.

### Dose of indocyanine green and different camera systems

Three different doses of ICG were used during this study. There was no positive correlation between ICG dose and the amount of transcutaneous detected SLNs. Table 4 gives an overview of the transcutaneous detection rate for all separate doses.

**Table 4:** Transcutaneous detection of the dissected sentinel lymph nodes for each dose

Transcutaneous detection	Yes *n*(%)	No *n*(%)	Total *n*
2.5 mg ICG	0 (0)	2 (100)	2
5.0 mg ICG	2 (22.2)	7 (77.8)	9
10 mg ICG	1 (14.3)	6 (85.7)	7
^99m^Tc-ICG dual tracer^a^	1 (12.5)	7 (87.5)	8
^99m^Tc-ICG dual tracer^a^ + 5.0 mg ICG	2 (40)	3 (60)	5
^99m^Tc-ICG dual tracer^a^ + 10 mg ICG	2 (25)	6 (75)	8
Total	7 (17.9)	32 (82.1)	39

ICG, indocyanine green; Tc, technetium.

a Dose is comparable with 0.01–0.04 mg of ICG.

As mentioned earlier, FLI was performed using either the Quest (*n* = 26), the Medtronic (*n* = 4) or a combination of both cameras (*n* = 2). The SLN was transcutaneously visible in five patients using the Quest (15.6%), in only one patient using the Medtronic (3.1%), and in none of the patients using both camera systems (0%).

## Discussion

The primary objective of the study was to evaluate the transcutaneous detection rate of SLNs with ICG for patients with melanoma. Transcutaneous SLN detection was achieved in 21.9% of patients, indicating that the exclusive use of FLI with ICG as a replacement for Tc in SLNB is currently not feasible. Furthermore, our study suggests that the use of different camera systems and adjustments in the dose of ICG are of no additional value.

Our findings suggest that a higher dose of ICG did not result in a higher transcutaneous detection rate, implying there is no detectable correlation between these two variables. Lese *et al*. [34] recently conducted a study similar to ours, as they also investigated the transcutaneous detection of SLNs in patients with melanoma using ICG. However, their results show significant discrepancies in our study, as they found a transcutaneous detection rate of 96.1%. Despite our efforts to replicate their methods, which included using the same dose of ICG and the same camera system, our transcutaneous detection rate was unable to reach similar levels. We explain this discrepancy through the difference in the definition of transcutaneous detection. Within the study conducted by Lese *et al*. [34], transcutaneous detection of the SLN was defined as any transcutaneous fluorescent signal identified using Visionsense VS3 HD3D camera. However, information is lacking regarding the spatial relationship between the incision and the transcutaneously detected fluorescent spot, as well as the detectability of the fluorescence signal after incision. These aspects are crucial for accurately determining the origin of the fluorescent signal, whether it originates from the SLN or is influenced by other factors such as autofluorescence or tracer spill. Therefore, we added these aspects to the definition of transcutaneous detection in our study.

Despite the low transcutaneous detection rate, our study did demonstrate a correlation between ICG and Tc in the context of SLN mapping. Out of the patients who received ICG and Tc separately and not the Tc-ICG dual tracer, 17 out of 18 nodes (94.4%) were both Tc positive and fluorescent. It is worth mentioning that, if there was no extra ICG signal after harvesting the SLN, no further attempt was made to search for ICG-positive lymph nodes, which could implicate that ICG-positive and Tc-negative nodes were missed. The robust association between Tc-positive lymph nodes and ICG-positive lymph nodes has also been demonstrated in previous studies [21,22], implying that ICG is a reliable tracer for finding SLNs in patients with melanoma.

Bargon *et al*. [21] administered ICG during SLNB in breast cancer patients in combination with a standard lower axillary incision, which resulted in only the use of FGS. Within breast cancer surgery, a standard incision is feasible due to the fact that 98.8–100% of the SLNs in breast cancer are routinely found in the same location within the axillary lymph node basin [35]. This is not the case with melanomas. In melanoma patients, the localization of the SLN varies not only between lymph node basins but also within these basins. For this reason, a standard incision is not feasible, even if the right lymph node basin is identified. Therefore, transcutaneous detection of the SLN would be of great value in patients with melanoma. The scope of this research does not include the preoperative use of FGS. Nevertheless, this is a subject that has the potential to be beneficial and demands additional research that is conducted in a systematic manner.

Although our study only reported a transcutaneous detection rate of ICG of 21.7%, most of the SLNs accumulated ICG. Therefore, if the transcutaneous detection increases, ICG could be a suitable tracer. To enhance the performance of transcutaneous detection of ICG, different alternate methodologies could be explored, such as photoacoustic imaging (PAI) or NIR-2 spectrum-sensitive camera systems [36,37]. The NIR-2 cameras are capable of detecting NIR fluorescence wavelengths above 900 nm. It is well established that longer wavelengths are associated with reduced light scattering within tissue, with the extent of scattering being dependent on the specific kind of tissue. Consequently, this phenomenon enables the light to penetrate deeper into the tissue, potentially reaching depths of up to 2 cm. As the emitted light from ICG exhibits a maximum wavelength of 1300 nm, the use of a NIR-2 camera might be a suitable alternative to increase the penetration depth during SLNB. Furthermore, the application of PAI in the identification of ICG is achieved by utilizing ultrasonic vibrations produced through laser-induced thermal expansion. This technique holds promise in providing a substantial detection depth of approximately 3.2 cm [38].

In conclusion, this study evaluated the use of ICG-fluorescence as a single tracer during SLNB as an alternative to Tc in patients with cutaneous melanomas, with the goal of eventually being able to completely eliminate Tc from the clinic, given its logistic challenges and higher costs. As we achieved a relatively low transcutaneous SLN detection rate, exclusive use of FLI with ICG is currently not yet feasible. However, we did find a high accumulation of ICG in the SLN (95%), indicating the potential of ICG in combination with other imaging techniques, such as PAI and NIR-2 fluorescence imaging.

## Acknowledgements

### Conflicts of interest

There are no conflicts of interest.
